# Monocytes in sarcoidosis are potent tumour necrosis factor producers and predict disease outcome

**DOI:** 10.1183/13993003.03468-2020

**Published:** 2021-07-22

**Authors:** Rico Lepzien, Sang Liu, Paulo Czarnewski, Mu Nie, Björn Österberg, Faezzah Baharom, Jamshid Pourazar, Gregory Rankin, Anders Eklund, Matteo Bottai, Susanna Kullberg, Anders Blomberg, Johan Grunewald, Anna Smed-Sörensen

**Affiliations:** 1 Division of Immunology and Allergy, Dept of Medicine Solna, Karolinska Institutet, Karolinska University Hospital, Stockholm, Sweden; 2 Dept of Biochemistry and Biophysics, National Bioinformatics Infrastructure Sweden, Science for Life Laboratory, Stockholm University, Stockholm, Sweden; 3 Dept of Public Health and Clinical Medicine, Division of Medicine, Umeå University, Umeå, Sweden; 4 Division of Respiratory Medicine, Dept of Medicine Solna and Centre for Molecular Medicine, Karolinska Institutet, Karolinska University Hospital, Stockholm, Sweden; 5 Dept of Respiratory Medicine, Theme Inflammation and Infection, Karolinska University Hospital, Stockholm, Sweden; 6 Division of Biostatistics, Institute of Environmental Medicine, Karolinska Institutet, Stockholm, Sweden

## Abstract

**Background:**

Pulmonary sarcoidosis is an inflammatory disease characterised by granuloma formation and heterogeneous clinical outcome. Tumour necrosis factor (TNF) is a pro-inflammatory cytokine contributing to granuloma formation and high levels of TNF have been shown to associate with progressive disease. Mononuclear phagocytes (MNPs) are potent producers of TNF and highly responsive to inflammation. In sarcoidosis, alveolar macrophages have been well studied. However, MNPs also include monocytes/monocyte-derived cells and dendritic cells, which are poorly studied in sarcoidosis, despite their central role in inflammation.

**Objective:**

To determine the role of pulmonary monocyte-derived cells and dendritic cells during sarcoidosis.

**Methods:**

We performed in-depth phenotypic, functional and transcriptomic analysis of MNP subsets from blood and bronchoalveolar lavage (BAL) fluid from 108 sarcoidosis patients and 30 healthy controls. We followed the clinical development of patients and assessed how the repertoire and function of MNP subsets at diagnosis correlated with 2-year disease outcome.

**Results:**

Monocytes/monocyte-derived cells were increased in blood and BAL of sarcoidosis patients compared to healthy controls. Interestingly, high frequencies of blood intermediate monocytes at time of diagnosis associated with chronic disease development. RNA sequencing analysis showed highly inflammatory MNPs in BAL of sarcoidosis patients. Furthermore, frequencies of BAL monocytes/monocyte-derived cells producing TNF without exogenous stimulation at time of diagnosis increased in patients that were followed longitudinally. In contrast to alveolar macrophages, the frequency of TNF-producing BAL monocytes/monocyte-derived cells at time of diagnosis was highest in sarcoidosis patients that developed progressive disease.

**Conclusion:**

Our data show that pulmonary monocytes/monocyte-derived cells are highly inflammatory and can be used as a predictor of disease outcome in sarcoidosis patients.

## Abstract 2

**Phenotypic, transcriptomic and functional mapping of blood and pulmonary mononuclear phagocytes in sarcoidosis patients found that frequency and function of pulmonary monocytes at time of diagnosis predict 2-year outcome in sarcoidosis** https://bit.ly/2JX8fhr

## Introduction

Sarcoidosis is a multisystemic T-cell driven inflammatory disorder of unknown aetiology with broad clinical heterogeneity. The hallmark of sarcoidosis is formation of granulomas that most commonly affect the lungs. Sarcoidosis resolves in 30–40% of the cases within 2 years [1–4]. In Sweden, one-third of sarcoidosis patients present with an acute disease onset, referred to as Löfgren's syndrome (LS) that associates with good prognosis. However, two-thirds of patients present with gradual-onset (non-LS) sarcoidosis and these patients are more likely to develop chronic disease [5]. What dictates disease severity is still unclear, but local immunological events early during disease development probably set the stage for disease progression, in addition to genetic and environmental factors [6].

T-cells have been best studied and T-cell driven mechanisms contributing to disease pathophysiology have been identified, while innate immune mechanisms in sarcoidosis are less well understood [6]. Several biological markers that predict disease severity, such as serum interleukin (IL)-2 receptor, serum amyloid A and tumour necrosis factor (TNF) have been identified, while candidates predicting disease outcome are still lacking [7, 8]. TNF is a multifunctional cytokine produced by a variety of cells and is important for the induction and maintenance of granulomas [9, 10]. *In vitro* cultured alveolar macrophages from sarcoidosis patients are major producers of TNF [11, 12]. In sarcoidosis, alveolar macrophages produce TNF without exogenous stimulation and highest levels of TNF are measured in patients with progressive disease [8]. Thus, drugs targeting TNF are used as a third-line treatment in non-LS patients, resulting in noticeable clinical improvement in a subgroup of the treated patients [13].

In addition to alveolar macrophages, monocytes/monocyte-derived cells and dendritic cells, collectively called mononuclear phagocytes (MNPs), are found lining the respiratory mucosa [14–16]. Pulmonary monocytes/monocyte-derived cells from healthy controls are potent producers of TNF upon stimulation *in vitro* [14]. However, functional data of pulmonary monocytes/monocyte-derived cells from sarcoidosis patients are currently not available. We previously reported no difference in the distribution of pulmonary monocytes/monocyte-derived cells from non-LS and LS patients, but comparison to pulmonary monocytes/monocyte-derived cells from healthy controls is still missing [17]. However, blood monocytes were shown to be affected in sarcoidosis. CD14^+^CD16^+^ intermediate monocytes, a subset of monocytes, are expanded in circulation of sarcoidosis patients, probably due to the systemic inflammation, as observed in other inflammatory diseases [18–21]. Furthermore, higher frequencies of circulating intermediate monocytes were associated in patients with response to anti-TNF treatment [22]. In contrast to monocytes, distribution of dendritic cells was not altered in blood or bronchoalveolar lavage (BAL) of sarcoidosis patients [23, 24].

In the present study, we aimed to determine to what extent pulmonary monocytes/monocyte-derived cells contribute to inflammation in sarcoidosis. In a detailed, longitudinal study, we show that monocytes/monocyte-derived cells actively contribute to inflammation by TNF production and associate with disease progression in non-LS sarcoidosis.

## Material and methods

### Study design and patient characteristics

The study was approved by the regional ethical review boards in Stockholm and Umeå, Sweden and performed according to the Declaration of Helsinki. 108 sarcoidosis patients and 30 healthy volunteers were included in the study and gave written informed consent (table 1). Bronchoscopies were performed at the Karolinska University Hospital, Stockholm, or at the University Hospital, Umeå, as described previously [17, 25]. All patients were newly diagnosed with pulmonary sarcoidosis as defined by World Association for Sarcoidosis and Other Granulomatous Disorders guidelines [26] based on clinical signs, chest radiography findings, an elevated CD4/CD8 T-cell ratio in BAL or noncaseating granulomas in tissue biopsies (table 1, supplementary figure S1a–c). 20 patients were diagnosed with LS based on clinical signs (acute disease onset, enlarged bilateral hilar lymph nodes, erythema nodosum and/or periarticular tendovaginitis).

**TABLE 1:** Clinical characteristics of healthy controls and sarcoidosis patients at time of diagnosis

	**Non-Löfgren's syndrome**	**Löfgren's syndrome**	**Healthy controls**
**Subjects**	88	20	30
**Male/female**	65/23	16/4	20/10
**Age years**	47 (38–56)	39 (36–44.5)	25 (24–30.5)
**Chest radiographic stage (0/I/II/III/IV)**^#^	1/18/41/13/2	0/9/10/0/0	N/A
**Smoking status (never-smoker/ex-smoker**^¶^**/smoker)**	60/22/5	7/10/3	25/0/5
**Extrapulmonary involvement (eye/spleen/kidney/skin)**	3/1/1/1	0	N/A
**Lung function % predicted**			
VC	94 (81.5–102) (n=54)	94 (86–102) (n=13)	N/A
*D*_LCO_	90 (81–103.5) (n=49)	93 (87.5–105) (n=10)	N/A
FEV_1_	86 (75.5–99) (n=53)	87 (81–98.5) (n=13)	102.6 (93.6–108.5)
**BAL fluid characteristics**			
Cell concentration ×10^6^ cells·L^−1^	155 (114–212) (n=81)	284 (122–405) (n=16)	29 (13–81)
Macrophages %	75 (61–83)	80 (60–87)	90 (85–93)
T-cells %	23 (14.5–36.5)	16 (11–36)	9 (5–13)
Neutrophils %	1.45 (0.8–3)	1.8 (1–3)	1.5 (0.6–2.4)
Eosinophils %	0.2 (0–1)	0.7 (0.4–1.6)	0.2 (0–0.6)
CD4/CD8 ratio	6.1 (3.5–8.7)	6.3 (3.65–7.8)	2.6 (1.7–4.8) (n=27)
Vα2.3^+^ CD4^+^ T-cells	4 (3.2–6.3) (n=81)	16.2 (2.9–29) (n=16)	N/D
**Serum ACE**	42 (25–60) (n=80)	45 (33–67) (n=19)	N/D
**Serum albumin**	38 (37–41) (n=86)	40 (37–42) (n=19)	40 (37–42) (n=15)
**2-year disease outcome (remission/stable/progressive)**	8/31/19	10/1/0	N/A

Data are presented as n or median (interquartile range). VC: vital capacity; *D*_LCO_: diffusing capacity of the lung for carbon monoxide; FEV_1_: forced expiratory volume in 1 s; BAL: bronchoalveolar lavage; ACE: angiotensin-converting enzyme; N/A: not applicable; N/D: not determined. ^#^: stage I: hilar/mediastinal lymph node enlargement; stage II: lymph node enlargement and lung parenchyma infiltrates; stage III: lung parenchyma infiltrates; stage IV: pulmonary fibrosis; ^¶^: smoke-free for >1 year.

In a cohort of nine non-LS patients, as part of a study with controlled exercise [27], a second bronchoscopy was performed 6 months after the first one to follow patients longitudinally (table 2).

**TABLE 2:** Clinical characteristics of a cohort of non-Löfgren's syndrome patients included in a controlled physical exercise study [27]

	**Exercise cohort**	
	**Time of diagnosis**	**Time of diagnosis +6** **months**
**Subjects n**	9	
**Male/female n/n**	7/2	
**Age years**	47.5	
**Chest radiographic stage (I/II/III/IV)**^#^	1/4/4/0	1/4/4/0
**Smoking status (never-smoker/ex-smoker**^¶^**/smoker)**	8/1/0	
**Lung function % predicted**		
TLC	88	89
*D*_LCO_	98	97
FEV_1_	90	87
**BAL fluid characteristics**		
Cell concentration ×10^6^ cells·L^−1^	152	215
Macrophages %	63	77
T-cells %	33	20
Neutrophils %	3.1	1.8
Eosinophils %	0.7	0.6
CD4/CD8 ratio	6.8	7.6

Data are presented as median at the two sampling time points. TLC: total lung capacity; *D*_LCO_: diffusing capacity of the lung for carbon monoxide; FEV_1_: forced expiratory volume in 1 s; BAL: bronchoalveolar lavage. ^#^: stage I: hilar/mediastinal lymph node enlargement; stage II: lymph node enlargement and lung parenchyma infiltrates; stage III: lung parenchyma infiltrates; stage IV: pulmonary fibrosis; ^¶^: smoke-free for >1 year.

Disease outcome was assessed 2 years after establishing diagnosis. In this study, 69 patients had passed the 2-year mark and were characterised with one of the following disease outcomes: 1) remission (no symptoms and no chest radiological signs); 2) chronic stable (stable pulmonary manifestations without deterioration with no signs of inflammatory activity in laboratory parameters; no or minor chest radiological changes compared to previous assessment, no systemic treatment required); 3) chronic progressive (deterioration of symptoms and impairment in chest radiological signs compared to previous assessment, systemic treatment required).

### Single-cell preparations from blood and BAL and flow cytometry

Blood and BAL were processed within 1 h after retrieval for further applications. Peripheral blood mononuclear cells (PBMCs) were isolated from blood using Ficoll density gradient centrifugation. BAL samples were kept on ice, filtered through a 100-μm nylon filter (Syntab Therapeutics, Würselen, Germany) and centrifuged at 400×*g* for 15 min before downstream application. Cell suspensions were incubated with LIVE/DEAD™ Fixable Aqua/Blue Dead Cell Stain Kit (Life Technologies, Waltham, MA, USA) and Fc receptors blocked using FcR block (Miltenyi, Bergisch Gladbach, Germany) followed by staining with antibodies against surface molecules (supplementary table S1). For intracellular staining, cells were fixed using the Foxp3/Transcription Factor Staining Buffer Set (Invitrogen, Waltham, MA, USA). Briefly, cells were fixed for 20 min at room temperature followed by staining with antibodies against intracellular molecules (supplementary table S1). Cells were analysed using an LSRII or LSR Fortessa flow cytometer (both BD, Franklin Lakes, NJ, USA) and data were analysed using FlowJo X software (BD).

### Fluorescence-activated cell sorting

For cell sorting, blood cells were enriched using the RosetteSep Human Monocyte Enrichment Cocktail (StemCell Technologies, Vancouver, BC, Canada). Blood and BAL cells were used within 1 h after retrieval from the study subjects and stained with a validated panel of antibodies (supplementary table S1). For cell sorting, FACS Aria Fusion or AriaIII (both BD) were used. Sorted cells were subsequently resuspended in Qiazol Lysis reagent (Qiagen, Venlo, the Netherlands) and stored at −80°C until further use.

### RNA isolation and sequencing

For detailed information on RNA isolation and RNA sequencing, please refer to the supplementary material.

### Stimulation of PBMCs and BAL cells

PBMCs and BAL cells were cultured at 1×10^6^ cells·mL^−1^ in RPMI 1640 (Merck, Darmstadt, Germany) containing 10% fetal calf serum (Invitrogen). Cells were cultured for 3 h either unstimulated or 1 μg·mL^−1^ lipopolysaccharide (LPS) (Merck; *Escherichia coli* O111:B4, L4391) was added. 10 μg·mL^−1^ brefeldin A (Merck) was added.

### ELISA

For detailed information on cytokine analysis using ELISA, please refer to the supplementary material.

### Statistical analysis

The numeric data are summarised with the median, unless otherwise stated. Statistical analyses were performed using the Mann–Whitney U-test, the nonparametric Kruskal–Wallis test with Dunn's test for correction of multiple comparisons, and the paired Wilcoxon signed-rank test. The Spearman's rank correlation coefficient was used for correlation analysis. Predictive models were estimated with logistic regression. The outcome variable was defined as remission *versus* no remission (chronic stable and chronic progressive) as well as chronic stable *versus* chronic progressive. The predictors entered the models one at a time. The categorical predictors were introduced by means of dummy variables. Data were analysed using GraphPad Prism version 8.0 (GraphPad Software, San Diego, CA, USA) and Stata (StataCorp, College Station, TX, USA). Results were considered statistically significant at the level p<0.05.

## Results

### CD14^+^CD16^+^monocytes/monocyte-derived cells are increased in blood and BAL of sarcoidosis patients

**FIGURE 1:**
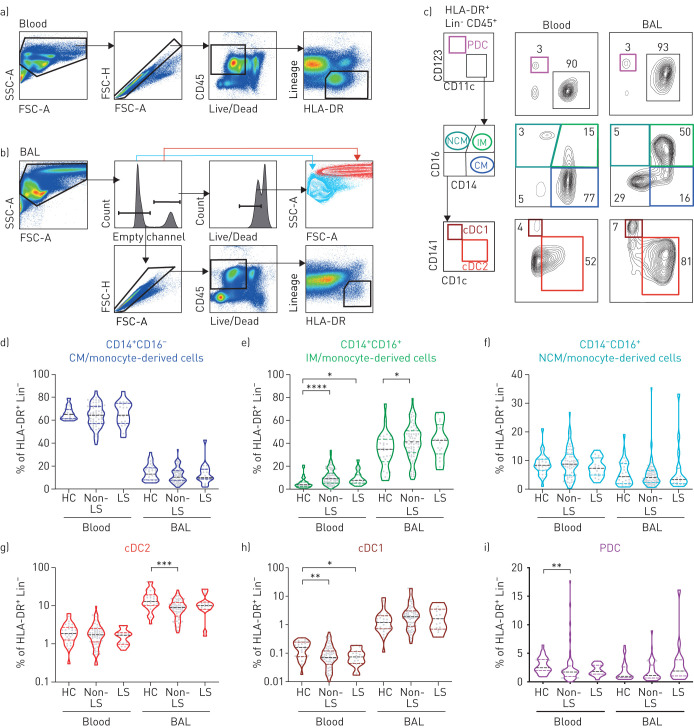
Elevated frequencies of monocytes/monocyte-derived cells in blood and bronchoalveolar lavage (BAL) of sarcoidosis patients compared to healthy controls. Pseudocolour plots from a) blood and b) BAL of one representative non-Löfgren's syndrome (LS) patient are shown to illustrate the gating strategy used to identify mononuclear phagocytes (MNPs). In BAL, alveolar macrophages were identified based on high autofluorescence and high side-scatter characteristics. Among single, live, CD45^+^ leukocytes, MNPs were identified expressing human leukocyte antigen (HLA)-DR but that were negative for lineage markers. c) Plasmacytoid dendritic cells (PDCs) were identified based on expression of CD123, while CD11c identified myeloid cells. From the CD11c^+^ cells, monocytes/monocyte-derived cells (CD14^+^CD16^−^ classical monocytes (CM), CD14^+^CD16^+^ intermediate monocytes (IM) and CD14^−^CD16^+^ nonclassical monocytes (NCM)) were identified. Subsequently, conventional dendritic cells (cDC)1 and cDC2 were identified by their expression of CD141 or CD1c, respectively. d–i) Violin plots show frequencies of d–f) monocyte and g–i) dendritic cell subsets out of live, HLA-DR^+^ lineage-negative cells in peripheral blood mononuclear cells and BAL in non-LS and LS sarcoidosis patients and healthy controls (HC). HC blood n=25, BAL n=28; non-LS blood n=64, BAL n=76; LS blood n=14, BAL n=16. Dotted lines indicate median, 25th and 75th percentile. Statistical analysis was performed using the non-parametric Kruskal–Wallis with Dunn's test for correction of multiple comparisons. *: p<0.05, **: p<0.01, ***: p<0.001, ****: p<0.0001.

To investigate the frequencies of MNP subsets in LS and non-LS sarcoidosis compared to healthy controls we performed multicolour flow cytometry on matched BAL and blood samples. In BAL, frequencies of alveolar macrophages, identified based on high side-scatter and autofluorescence, were reduced in non-LS and LS patients compared to healthy controls (figure 1b and supplementary figure S1d). In blood and BAL, MNPs were identified as human leukocyte antigen (HLA)-DR^+^ and lineage negative (figure 1a–b and supplementary figure S1e and f). Furthermore, three monocyte subsets as well as plasmacytoid dendritic cells (PDC) and conventional dendritic cells (cDC)1 and cDC2 were identified (figure 1c). Overall, distribution of MNP subsets differed most distinctly based on their origin from blood or BAL (figure 1d–i and supplementary figure S2a and b). Still, significant differences were observed in CD14^+^CD16^+^ intermediate monocytes, that were increased in blood of non-LS and LS patients and in BAL of non-LS patients compared to healthy controls (figure 1e). cDC2 and cDC1 were significantly decreased in BAL and blood, respectively, of non-LS patients compared to healthy controls (figure 1g and h). With respect to maturation status of MNPs, the most apparent difference was that BAL MNPs were more mature than blood MNPs by upregulation of HLA-DR and CD86. However, MNPs from non-LS patients in blood and BAL expressed higher levels of HLA-DR and CD86 compared to those from healthy controls within each anatomical compartment (supplementary figure S2c and d). In summary, based on flow cytometric analysis, we documented limited but potentially important alterations in the frequency and maturation of dendritic cells and monocytes/monocyte-derived cells in BAL and blood in sarcoidosis patients.

### MNPs from sarcoidosis patients present inflammatory gene signature

**FIGURE 2:**
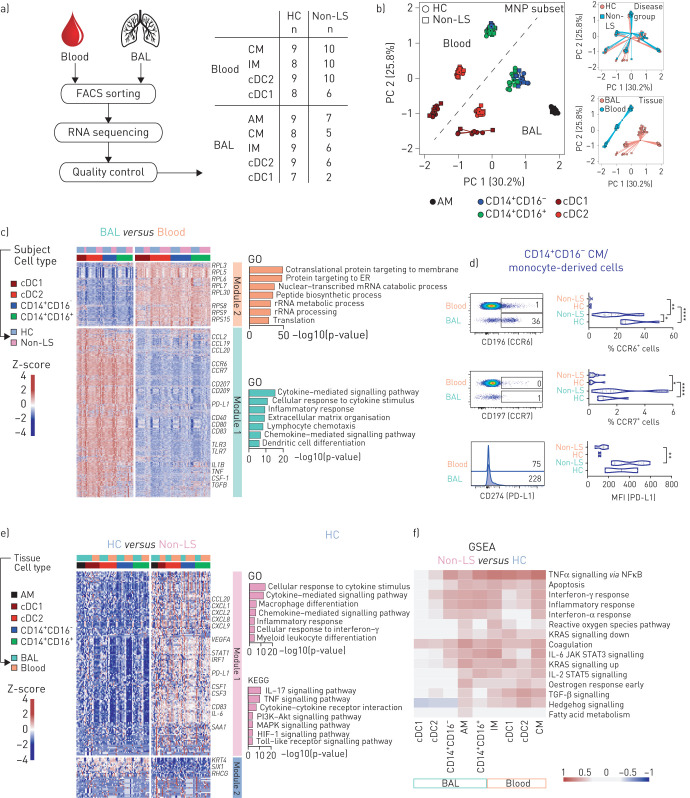
Pro-inflammatory gene sets enriched in mononuclear phagocytes (MNPs) from sarcoidosis patients compared to healthy controls. a) Overview shows the workflow for samples used for RNA sequencing. Table shows number of samples of MNP subsets from healthy controls (HC) and non-Löfgren's syndrome (LS) patients that passed quality control for further analysis. b) Principal component (PC) analysis of normalised and batch-corrected read counts based on MNP subset, sex and tissue. The percentage of variance explained by the respective principal component is indicated in parenthesis. c) Heatmap shows differentially expressed genes in MNPs comparing blood and bronchoalveolar lavage (BAL). Module 1 contains genes with higher and module 2 with lower gene expression in BAL compared to blood. Selected genes are highlighted and annotated to gene ontology (GO) terms. d) Validation of differential gene expression genes for CCR6 (HC n=5, non-LS n=17), CCR7 (HC n=6, non-LS n=19) shown as percentage of classical monocytes (CM), and programmed death ligand 1 (PD-L1) (HC n=2, non-LS n=6) shown as median fluorescence intensity (MFI) in CD14^+^CD16^−^ CM/monocyte-derived cells from blood and BAL in HC and non-LS patients. Statistical analysis was performed using the nonparametric Mann–Whitney U unpaired t-test. *: p<0.05, **: p<0.01, ****: p<0.0001. e) Heatmap shows differentially expressed genes in MNPs comparing HC and non-LS patients. Module 1 contains genes with higher expression and module 2 genes with lower expression in non-LS patients compared to HC. Selected genes are highlighted and annotated to GO terms and Kyoto Encyclopedia of Genes and Genomes (KEGG) pathways. f) Heatmap shows gene set enrichment analysis (GSEA) of differentially expressed genes with annotated hallmark genes comparing MNP subsets from blood and BAL of non-LS patients with HC. FACS: fluorescence-activated cell sorting; IM: intermediate monocytes; cDC: conventional dendritic cells; AM: alveolar macrophages.

To further examine MNPs in sarcoidosis, we sorted blood and BAL dendritic cells and monocytes/monocyte-derived cells as well as alveolar macrophages from BAL of non-LS patients and healthy controls and performed RNA sequencing on in total nine different populations (figure 2a). Principal component analysis showed that samples distributed based on tissue source and cell subset rather than their origin from non-LS patients or healthy controls (figure 2b). We found that genes were upregulated in BAL MNPs related to cell maturation (*CD40*, *CD80*, *CD83*), inflammatory response (*TLR3*, *TLR7*), cytokine signalling (*TNF*, *IL1B*, *CSF1*, *TGFβ*) and chemotaxis (*CCR6*, *CCR7* as well as *CCL2*, *19* and *20*) compared to blood MNPs in both non-LS patients and healthy controls (figure 2c). The difference in mRNA expression of *CCR6*, *CCR7*, *CD207* and *PD-L1*, all critical for the function of MNPs, was confirmed on protein level (figure 2d and supplementary figure S2c–e). Importantly, we next analysed gene signatures across cell subsets comparing non-LS patients and healthy controls. We observed upregulation of genes related to TNF, IL-17 and Toll-like receptor (TLR) signalling (figure 2e) in non-LS patients compared to healthy controls. Gene set enrichment analysis of each sequenced MNP subset verified high expression of genes related to the TNF pathway (such as *TNF*, *IL1B*, *IL6*, *NR4A1* and *REL*) as well as other immune-related pathways in samples from non-LS patients compared to healthy controls (figure 2f). Taken together, utilising RNA sequencing revealed distinct differences in cytokine signalling across MNP subsets upregulated in non-LS patients compared to healthy controls, in particular related to TNF signalling.

### Monocytes/monocyte-derived cells from non-LS patients show high TNF gene and protein expression at time of diagnosis and during disease development

**FIGURE 3:**
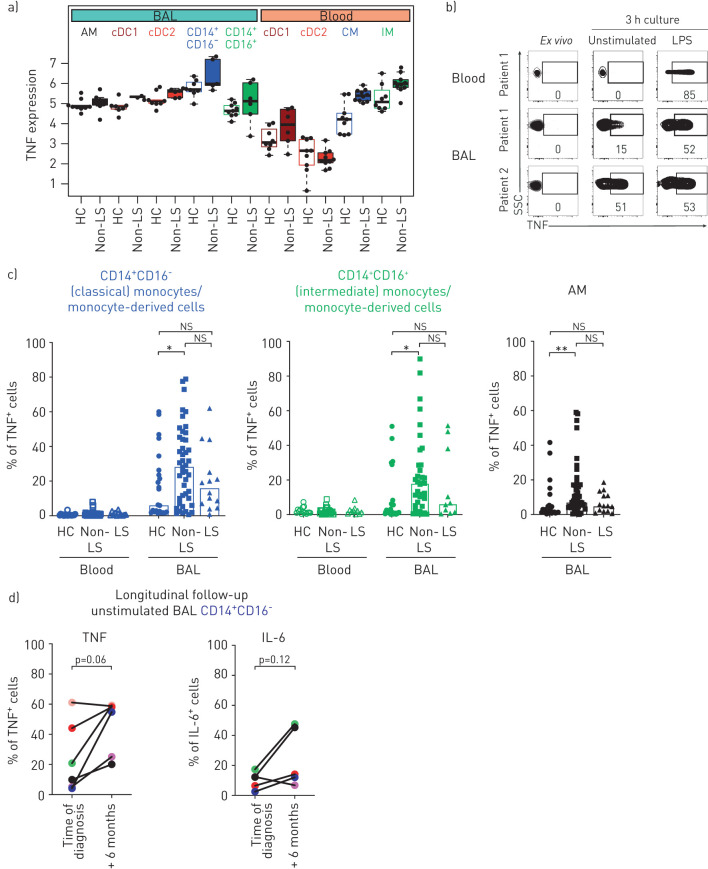
Monocytes/monocyte-derived cells from bronchoalveolar lavage (BAL) of sarcoidosis patients with excessive tumour necrosis factor (TNF) production without stimulation. a) Graph shows the normalised relative *TNF* gene count from blood and BAL of mononuclear phagocytes (MNPs) in healthy controls (HC) (open bars) and non-Löfgren's syndrome (LS) patients (filled bars). b) Contour plots show intracellular TNF staining in CD14^+^CD16^−^ classical monocytes/monocyte-derived cells from blood and BAL of two non-LS patients stained *ex vivo* and after 3 h culture without stimulation and after adding lipopolysaccharide (LPS) (1 μg·mL^−1^) in the presence of brefeldin A (10 μg·mL^−1^). c) Bar graphs show the frequency of TNF-expressing CD14^+^CD16^−^ and CD14^+^CD16^+^ monocytes/monocyte-derived cells and alveolar macrophages (AM) from blood and BAL of HC (blood n=17, BAL n=23), non-LS (blood+BAL n=39) and LS patients (blood n=12, BAL n=14) after 3 h culture without stimulation. Bars indicate the median. d) Graphs show percentage of TNF and interleukin (IL)-6 expressing CD14^+^CD16^−^ monocytes/monocyte-derived cells in BAL from non-LS patients after 3 h culture without stimulation at time of diagnosis and after 6 months. Colour coding identifies individual patients and lines connect each patient at time of diagnosis and after 6 months. Statistical analyses were performed using the nonparametric Kruskal–Wallis with Dunn's test for correction of multiple comparisons and the nonparametric paired Wilcoxon signed-rank test. ns: nonsignificant. *: p<0.05, **: p<0.01.

Next, we analysed whether the enriched TNF signalling pathway in individual MNP subsets from non-LS patients had functional implications. The relative expression of *TNF* gene transcripts in MNPs was overall higher in BAL MNPs compared to blood (figure 3a). Furthermore, *TNF* in each subset from non-LS patients was higher compared to healthy controls MNPs (figure 3a). To confirm this, we measured TNF protein in individual MNPs using intracellular cytokine staining and flow cytometry, either *ex vivo* or after 3 h of culture with and without stimulation (figure 3b). In blood of all subjects, TNF could only be detected after LPS stimulation in monocytes (figure 3b and supplementary figure S4a). In contrast, BAL monocytes/monocyte-derived cells showed an accumulation of TNF intracellularly without stimulation (figure 3b and c). In some patients, adding LPS did not result in higher frequency of TNF-expressing cells than the unstimulated condition (supplementary figure S4a). The frequency of unstimulated TNF-expressing MNPs was significantly higher in non-LS patients compared to healthy controls and LS patients (figure 3c). The frequency of unstimulated TNF expressing monocytes/monocyte-derived cells was higher compared to dendritic cells (supplementary figure S4b). Higher frequencies of TNF-producing monocytes/monocyte-derived cells correlated positively with higher frequencies of TNF-producing macrophages or dendritic cells of the same patient (supplementary figure S4c). TNF was detectable in plasma and BAL fluid; however, no correlations were observed between secreted TNF and the frequency of unstimulated TNF-expressing BAL monocytes/monocyte-derived cells (supplementary figure S4d and e).

The IL-6 signalling pathway was also enriched in the RNA sequencing analysis of MNPs from non-LS patients and we confirmed similar transcriptional and protein expression pattern for IL-6, as seen for TNF (supplementary figure S5a–f). LS patients showed a trend towards higher frequencies of IL-6 expression in unstimulated BAL MNPs compared to non-LS patients and healthy controls. Frequencies of TNF- and IL-6-expressing unstimulated BAL monocytes/monocyte-derived cells correlated positively in healthy controls and non-LS patients, but showed a trend towards negative correlation in LS patients (supplementary figure S5g). In addition, IL-1β was significantly increased in plasma, but not BAL fluid in non-LS patients compared to healthy controls or LS patients (supplementary figure S6).

To assess whether the elevated TNF-producing MNPs in BAL was maintained during disease development, we analysed samples from a study where newly diagnosed sarcoidosis patients followed a controlled physical exercise programme but did not receive any treatment [27]. We observed that frequencies of TNF-expressing unstimulated MNPs from non-LS patients increased over time (figure 3d). Frequency of IL-6 expressing MNPs as well as MNP distribution were not altered during this time (figure 3d and supplementary figure S7a–c).

Our data suggest that elevated frequencies of TNF-producing monocytes/monocyte-derived cells in BAL, without additional stimulation, mark an important immunological difference between non-LS patients at time of diagnosis from both LS patients and healthy controls.

### Monocytes/monocyte-derived cells and dendritic cells are predictors of the disease outcome in sarcoidosis

**FIGURE 4:**
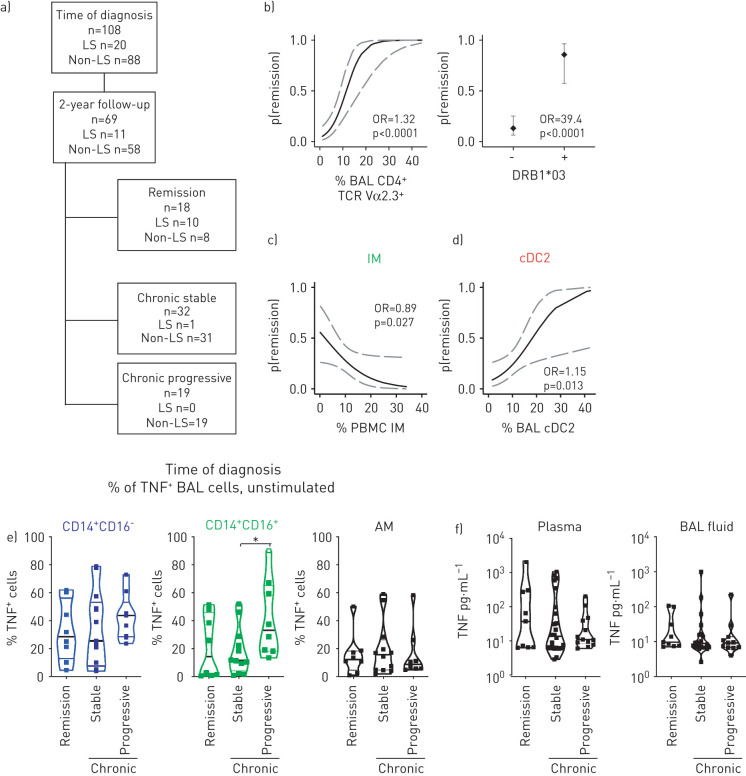
High frequencies of monocytes and unstimulated tumour necrosis factor (TNF) expression indicate disease progression in non-Löfgren's syndrome (LS) sarcoidosis. a) Overview of included patients at time of diagnosis and follow-up after 2 years with having either remitted disease or developed chronic stable or chronic progressive disease. b) Plots show predictive modelling of the general experimental parameters Vα2.3^+^ T-cells in sarcoidosis patients and HLA-DRB1*03 status. Plots show the probability (p) to clear the disease within 2 years after establishing diagnosis. Dotted lines indicate the 95% confidence interval. c,d) Plots show predictive modelling of c) CD14^+^CD16^+^ intermediate monocytes (IM) in blood and d) conventional dendritic cells (cDC)2 in bronchoalveolar lavage (BAL) of sarcoidosis patients. Dotted lines indicate the 95% confidence interval. e,f) Violin plots show e) TNF expression after 3 h culture without stimulation by CD14^+^CD16^−^ and CD14^+^CD16^+^ monocytes/monocyte-derived cells, and alveolar macrophages (AM) at time of diagnosis and f) TNF concentrations in plasma and BAL fluid based on the outcome. Statistical analyses were performed using the nonparametric Kruskal–Wallis with Dunn's test for correction of multiple comparisons and predictive modelling using linear regression. PBMCs: peripheral blood mononuclear cells. *: p<0.05.

To determine whether MNP phenotype, cytokine levels or mRNA expression at time of diagnosis associated with two-year disease outcome we used predictive modelling. Out of the 108 patients included in the study, 69 passed the 2-year mark at the time of analysis. 18 patients showed remission while 51 had developed chronic disease: 32 patients being chronic stable and 19 patients with chronic progressive disease (figure 4a). In line with previous reports [28], the probability to remit disease 2 years after diagnosis was higher (OR 1.32) when the frequency of Vα2.3^+^ T-cells was high in BAL as well as when patients were carrying the HLA-DRB1*03 allele (OR 39.4), which was mostly attributed to LS patients (figure 4b). Interestingly, patients with higher frequencies of CD14^+^CD16^+^ intermediate monocytes in blood at time of diagnosis were less likely to show remission after 2 years (OR 0.89) (figure 4c). In contrast, higher frequencies of cDC2 in BAL at time of diagnosis indicate a higher chance to remit disease after 2 years (OR 1.15) (figure 4d). Additionally, patients with higher frequencies of cDC1 (OR 0.54) and PDCs (OR 1.34) in BAL were more likely to not develop progressive disease or clear disease after 2 years, respectively (supplementary figure S8a).

In our dataset, TNF expression by alveolar macrophages and CD14^+^CD16^−^ monocytes/monocyte-derived cells did not predict disease outcome; however, TNF-expressing unstimulated CD14^+^CD16^+^ monocytes/monocyte-derived cells at time of diagnosis predicted a progressive disease development (figure 4e). Neither soluble TNF and IL-6 concentrations in plasma and BAL fluid nor IL-6-producing MNPs from BAL of sarcoidosis patients predicted disease outcome (figure 4f and supplementary figure S8b and c).

Collectively, our data show that monocytes/monocyte-derived cells are affected by and contribute actively to inflammation in non-LS patients. Furthermore, assessing monocytes/monocyte-derived cells at time of diagnosis can predict the disease course in non-LS sarcoidosis.

## Discussion

MNPs are likely to be central in sarcoidosis pathogenesis as they activate T-cells and produce pro-inflammatory cytokines that drive inflammation in sarcoidosis [29]. In the current study, we revealed that the frequency and distribution of monocytes and dendritic cells in blood and BAL at time of diagnosis may predict disease outcome. Furthermore, monocytes/monocyte-derived cells were potent TNF producers contributing to local and systemic inflammation and high frequencies of TNF producing monocytes/monocyte-derived cells associated with progressive disease development in sarcoidosis.

CD14^+^CD16^+^ intermediate monocytes are indicative of systemic inflammation and are elevated in several inflammatory diseases [20, 21]. In addition, we and others have seen elevated frequencies of intermediate monocytes in non-LS sarcoidosis patients [18, 19, 30–32]. Intermediate monocytes differentiate from CD14^+^CD16^−^ classical monocytes in blood and the process is accelerated during inflammatory conditions [33]. Cytokines in blood probably drive that differentiation. Reports on elevated plasma concentrations of TNF or IL-6 could not be confirmed in our cohort (supplementary figures S4d and S5f) [34, 35]. However, other cytokines may contribute to inflammation. As our RNA sequencing data indicated, *IL-1β* or *CSF-1* genes were upregulated in BAL MNPs that could be released into circulation and contribute to inflammation resulting in the increase of CD14^+^CD16^+^ intermediate monocytes. Increased expression of *IL-1β* by BAL MNPs indicates an upregulation of the inflammatory NLRP3 pathway as previously shown to be activated in sarcoidosis [36]. In BAL, increased levels of TNF and IL-6 were observed on both gene expression and protein levels compared to healthy controls indicating local inflammation and may explain the expansion of CD14^+^CD16^+^ monocytes/monocyte-derived cells in the lung of non-LS patients. Pro-inflammatory cytokines in the lung of sarcoidosis patients were observed before [37]. Another explanation for the expansion of CD14^+^CD16^+^ monocytes/monocyte-derived cells in BAL could be increased migration of CD14^+^CD16^−^ classical monocytes to the lung that subsequently differentiate to CD14^+^CD16^+^ monocytes/monocyte-derived cells. RNA sequencing data showed increased *CCL2* gene expression by BAL MNPs. CCL2 is the ligand for CCR2, which is highly expressed on CD14^+^CD16^−^ classical monocytes. Upon migration to the lung, the differentiation of CD14^+^CD16^−^ into CD14^+^CD16^+^ monocytes/monocyte-derived cells is known to be influenced by the tissue environment [33, 38]. Due to the younger age of the healthy control cohort, we cannot rule out that age-related effects influence the presence of CD14^+^CD16^+^ monocytes/monocyte-derived cells in non-LS patients compared with healthy controls.

In the current study, we show that pulmonary monocytes/monocyte-derived cells themselves contribute significantly to inflammation by production of pro-inflammatory TNF. We have previously shown that pulmonary monocytes/monocyte-derived cells could respond to TLR stimulation by producing TNF [14]. However, here we found high frequencies of TNF producing pulmonary monocytes/monocyte-derived cells even without stimulation. Our data complement the finding that in addition to alveolar macrophages as the principal producer of TNF in sarcoidosis patients [11], other TNF-producing immune cells contribute to inflammation in sarcoidosis. Most reports studying monocytes in sarcoidosis have been limited to blood. A study on blood monocytes showed a decrease in IL-10-producing regulatory monocytes in sarcoidosis patients compared with healthy controls, further strengthening the pro-inflammatory role of monocytes [39]. Less IL-10 produced by monocytes resulted in impaired suppression of T-cell proliferation, possibly contributing to the exaggerated T-cell alveolitis observed in sarcoidosis [39]. Additionally, blood monocytes from sarcoidosis patients responded with higher TNF and IL-6 production after stimulation compared with controls [18]. This supports our RNA sequencing data that blood monocytes are highly inflammatory although to a different extent as BAL monocytes/monocyte-derived cells that released TNF spontaneously. While alveolar macrophages are numerous in BAL fluid and undoubtedly play an important role in sarcoidosis, analysis of bulk BAL cells may mask the contribution of less frequent monocytes/monocyte-derived cells and dendritic cells [40]. By studying the transcriptome of individual MNP subsets, we could also identify differences in gene expression between alveolar macrophages, monocytes/monocyte-derived cells and dendritic cells (figure 2f). Alveolar macrophages showed upregulated genes related to the reactive oxygen species pathway, transforming growth factor (TGF)-β or the fatty acid metabolism. Interestingly, in BAL of non-LS patients, colony-stimulating factor-1 was upregulated by MNPs that favour the differentiation of alternatively activated macrophages [41]. In support of this, MNPs in BAL of non-LS patients also express high levels of TGF-β compared to controls, also favouring alternatively activated macrophage polarisation [42]. Potentially, single-cell RNA sequencing could better reveal the heterogeneity of classically and alternatively activated alveolar macrophages in sarcoidosis. It is likely that the balance between pro- and anti-inflammatory macrophages contribute to disease progression and resolution. Collectively, these findings mark significant differences between alveolar macrophages and monocytes/monocyte-derived cells in the lungs of sarcoidosis patients.

Serum amyloid A (SAA) and soluble IL-2 receptor were markers found in patients with progressive disease [7, 8]. Our RNA data confirmed that SAA was increased non-LS patients compared to healthy controls. In addition to disease severity markers, prognostic factors are needed to determine disease outcome of the patients that a deterioration of symptoms can be intervened early on. We found that high frequencies of blood monocytes and lower frequencies of BAL dendritic cells at time of diagnosis are predictors of disease outcome as patients were less likely to resolve disease after 2 years. To assess whether frequencies of monocytes can be used as a predictive tool in the clinic, a prospective multicentre study would be desirable to obtain larger patient numbers to account for differences in location, age, sex and ethnicity. Functionally, unstimulated TNF-producing BAL monocytes/monocyte-derived cells, but not alveolar macrophages, predicted chronic progressive disease development. This is in complementing previous observations where TNF production by alveolar macrophages were observed in patients with progressive disease [8]. A strength of our study is the use of flow cytometry and intracellular cytokine staining that allows identification of TNF expression on a single-cell level compared to ELISA that measures total secreted TNF without revealing the source of the cytokine production. Additionally, we used a short 3-h culture for TNF to accumulate with the advantage to primarily induce cytokine production in MNPs rather than T-cells. Since the frequency of CD14^+^CD16^+^ intermediate monocytes at time of diagnosis correlates with a chronic disease course after 2 years, it is of interest whether inhibition or modulation of monocyte migration by targeting the CCR2-CCL2 signalling axis could be explored as new treatment targets [8, 43].

Collectively, our data reveal an important role for monocytes/monocyte-derived cells in sarcoidosis. While monocytes are pro-inflammatory, alveolar macrophages are important for regeneration as well as tissue repair and dendritic cells most likely are essential in the T-cell response that has to be elucidated in further studies. Continued in-depth analysis of MNPs may help to better understand the clinical heterogeneity and may pave the way to identify biomarkers and treatment options to help sarcoidosis patients clear the disease.

## Supplementary material

10.1183/13993003.03468-2020.Supp1 **Please note:** supplementary material is not edited by the Editorial Office, and is uploaded as it has been supplied by the author.
Supplementary material ERJ-03468-2020.SUPPLEMENT

## Shareable PDF

10.1183/13993003.03468-2020.Shareable1 This one-page PDF can be shared freely online.
Shareable PDF ERJ-03468-2020.Shareable
