# Supplementary material for: Monocytes in sarcoidosis are potent tumour necrosis factor producers and predict disease outcome
Source: Eur Respir J. 2021 Jul 22;58(1):2003468. doi: 10.1183/13993003.03468-2020 (PMC8295505; doi:10.1183/13993003.03468-2020)
Supplement: Supplementary file 1 [file ERJ-03468-2020.SUPPLEMENT.pdf]

Monocytes in sarcoidosis are potent TNF producers and predict disease outcome

Rico Lepzien, Sang Liu, Paulo Czarnewski, Mu Nie, Björn Österberg, Faezzah Baharom, Jamshid Pourazar, Gregory Rankin, Anders Eklund, Matteo Bottai, Susanna Kullberg, Anders Blomberg, Johan Grunewald, and Anna Smed-Sörensen

ONLINE DATA SUPPLEMENT

Methods Supplement

RNA isolation and sequencing

RNA was isolated using the miRNeasy micro kit (Qiagen) according to the manufacturer's protocol. Briefly, chloroform was added to cell homogenates and centrifuged. The aqueous phase was mixed with 1.5 volumes of 100% ethanol. The samples were transferred to a RNeasy MinElute spin column and centrifuged. After, the column was washed with RPE buffer and spun down followed by addition of 80% ethanol and centrifugation. RNA was eluted in a collection tube using RNAse-free water. RNA quantity and quality were assessed using a bioanalyzer and samples with an RNA integrity number > 6 used for further application. Library preparation was performed by Novogene, using TruSeq Stranded mRNA Library Prep Kit (poly-A selection*)* when the total RNA amount was more than 100ng, if the sample total RNA amount was lower than 100ng, the library preparation was done with SMART-Seq v4 Ultra Low Input RNA Kit for Sequencing (Clontech). Sequencing was performed using a HiSeq-4000 platform (Illumina) with a 2x150-bp pair-end sequencing setup and the sequencing depth was 20 M reads/sample.

RNA-seq data analysis

Pair-ended reads were evaluated for sequencing quality using FASTQC, were trimmed for adaptor sequences and aligned using TopHat2 [1] with HGC38 human genome. Further analysis was then performed in R. Counts were summed up to the gene level and focusing the analysis of protein-coding genes using Biomart's [2] transcript annotation from Ensembl. Genes detected in fewer than 2 samples with counts per million (CPM) values lower than 1 were considered lowly expressed and

removed from the analysis. Unsupervised analysis and visualization of results were done using principal component analysis (PCA) on scaled and centered gene expression and agglomerative hierarchical clustering (HC) using Pearson correlation as distance matrix and “ward.D2” as linkage method. We observed that the library preparation method used for RNAseq influenced sample-wise relative log gene expression (Figure S3A), resulting in a general shift in gene expression as samples separated in PC1 by the library preparation method (Figure S3B). This was corrected using ComBat from sva package [3] preserving sorted cell grouping. Differential expression using EdgeR package [4] was done in several occasions depending on the comparison in question. A model depicting library preparation method, gender, disease group, tissue source and cell type was done to evaluate overall trends in gene expression (y) across those conditions:

$$y \sim LibraryPrep + Gender + DiseaseGroup + TissueSource + CellType \quad \text{(EQ.1)}$$

At a second instance, pair-wise comparisons in EdgeR were done to individually test the effect of disease status (non-LS versus HC) on each cell type per tissue (EQ.2):

$$y \sim DiseaseGroup \quad \text{(EQ.2)}$$

Similarly, pair-wise comparisons were done to test the effect of the tissue (blood vs. lung) on each cell type per disease status (EQ.3):

$$y \sim TissueSource \quad \text{(EQ.3)}$$

In all those comparisons above (EQ.1, 2 and 3), genes with fold change (FC) above 2 and false discovery rate (FDR) below 1e-4 were considered significant.

Functional analysis of differentially expressed genes (sorted by fold change) was done with EnrichR package [5] for gene list hypergeometric test (results from EQ.1) or with gene set enrichment analysis (GSEA) [6] using the fGSEA package [7] for each of the pair-wise comparisons described above (results from EQ.2 and EQ.3). The Gene

Ontology (GO), the Kyoto Encyclopedia of Genes and Genomes (KEGG) and the list of hallmark gene set from the Molecular Signatures Database (MSigDB) were used for enrichment testing.

ELISA

For detection of TNF, IL-6, and IL-1β in plasma and BAL fluid, the human TNF, IL-6, and IL-1β DuoSet ELISA (all R&D Systems) were used according to the manufacturer's instructions. Briefly, plates were coated with capture antibodies and incubated overnight. Plates were washed 4 times with wash buffer (PBS + 0.05% Tween 20) followed by blocking with reagent diluent for one hour at room temperature (RT). Subsequently, undiluted samples and serial dilution of standard were added and incubated for 2h at RT. Prior to the incubation, BAL fluid was concentrated 20x using Amicon Ultra-4 Centrifugal Filter Units (Merck). Next, the detection antibody was added and incubated for 2h at RT followed by incubation with Streptavidin-HRP for 20 min at RT and color reagent A + B (mixed 1:1) for 20 min at RT before adding stop solution and analysis on an ELISA reader. OD values were measured at 450nm and concentrations interpolated from the standard curve.

References

1. Kim D, Pertea G, Trapnell C, Pimentel H, Kelley R, Salzberg SL. TopHat2: accurate alignment of transcriptomes in the presence of insertions, deletions and gene fusions. *Genome biology* 2013: 14(4): R36.
2. Durinck S, Spellman PT, Birney E, Huber W. Mapping identifiers for the integration of genomic datasets with the R/Bioconductor package biomaRt. *Nature protocols* 2009: 4(8): 1184-1191.
3. Leek JT, Johnson WE, Parker HS, Jaffe AE, Storey JD. The sva package for removing batch effects and other unwanted variation in high-throughput experiments. *Bioinformatics (Oxford, England)* 2012: 28(6): 882-883.
4. Robinson MD, McCarthy DJ, Smyth GK. edgeR: a Bioconductor package for differential expression analysis of digital gene expression data. *Bioinformatics (Oxford, England)* 2010: 26(1): 139-140.
5. Kuleshov MV, Jones MR, Rouillard AD, Fernandez NF, Duan Q, Wang Z, Koplev S, Jenkins SL, Jagodnik KM, Lachmann A, McDermott MG, Monteiro CD, Gundersen GW, Ma'ayan A. Enrichr: a comprehensive gene set enrichment analysis web server 2016 update. *Nucleic acids research* 2016: 44(W1): W90-97.
6. Subramanian A, Tamayo P, Mootha VK, Mukherjee S, Ebert BL, Gillette MA, Paulovich A, Pomeroy SL, Golub TR, Lander ES, Mesirov JP. Gene set enrichment analysis: a knowledge-based approach for interpreting genome-wide expression profiles. *Proceedings of the National Academy of Sciences of the United States of America* 2005: 102(43): 15545-15550.
7. Korotkevich G, Sukhov V, Sergushichev A. Fast gene set enrichment analysis. *bioRxiv* 2019: 060012.

Supplementary table I. Antibodies used for surface and intracellular staining.

**Antibodies**	**Source**	**Identifier**
Surface antibodies		
anti-CD1c FITC human (clone AD5-8E7)	Miltenyi	130-090-507
anti-CD1c PE-Vio770 human (clone AD5-8E7)	Miltenyi	130-110-538
anti-CD3 APC-Cy7 human (clone SK7)	BD	341090
anti-CD3 FITC human (clone UCHT1)	BD	555332
anti-CD11c APC human (clone S-HCL-3)	BD	333144
anti-CD11c V450 human (clone B-Ly6)	BD	560369
anti-CD14 BV510 human (clone M5E2)	BioLegend	301842
anti-CD14 PE human (clone MoP9)	BD	359412
anti-CD16 AF700 human (clone 3G8)	BioLegend	302026
anti-CD19 APC-Cy7 human (clone HIB19)	BioLegend	302218
anti-CD19 FITC human (clone HIB19)	BioLegend	302205
anti-CD20 APC-Cy7 human (clone L27)	BD	335829
anti-CD20 FITC human (clone 2H7)	BD	555622
anti-CD45 APC human (clone HI30)	BD	555485
anti-CD56 APC-Cy7 human (clone HCD56)	BioLegend	318332
anti-CD56 FITC human (clone HCD56)	BioLegend	318304
anti-CD66abce APC-Vio770 human (clone TET2)	Miltenyi	130-119-847
anti-CD66abce FITC human (clone TET2)	Miltenyi	130-116-522
anti-CD86 BV650 human (clone FUN-1)	BD	563412
anti-CD123 PerCP-Cy5.5 human (clone 7G3)	BD	558714
anti-CD141 PE human (clone AD5-14H12)	Miltenyi	130-113-318
anti-CD141 VioBlue human (clone AD5-14H12)	Miltenyi	130-113-320
anti-HLA-DR APC-Cy7 human (clone L243)	BD	335831
anti-HLA-DR PE-Cy5 human (clone G46-6)	BD	555813
anti-HLA-DR PE-TR human (clone TU36)	Life Technologies	MHLDR17
Intracellular antibodies		
anti-IL-6 FITC human (clone MQ2-13A5)	BioLegend	551104
anti-TNF APC human (clone MAb11)	BD	554514

Figure S1

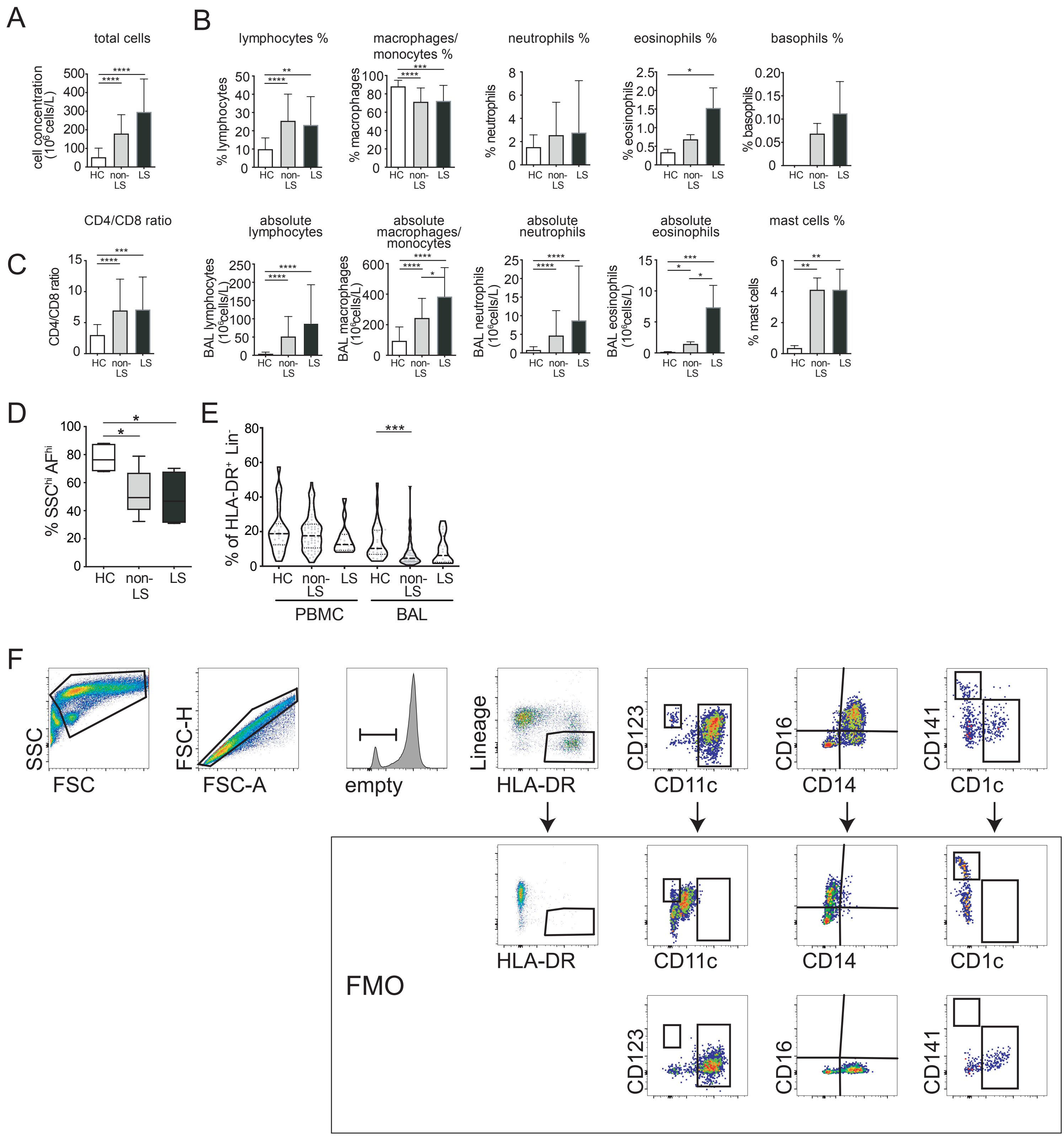

A
B
C
D
E
F
total cells
cell concentration (10^6 cells/L)
lymphocytes %
% lymphocytes
macrophages/ monocytes %
% macrophages
neutrophils %
% neutrophils
eosinophils %
% eosinophils
basophils %
% basophils
CD4/CD8 ratio
absolute lymphocytes
BAL lymphocytes (10^6cells/L)
absolute macrophages/ monocytes
BAL macrophages (10^6cells/L)
absolute neutrophils
BAL neutrophils (10^6cells/L)
absolute eosinophils
BAL eosinophils (10^6cells/L)
mast cells %
% mast cells
HC
non-LS
LS
% SSC^hi AF^hi
% of HLA-DR+ Lin-
PBMC
BAL
SSC
FSC
FSC-H
FSC-A
empty
Lineage
HLA-DR
CD123
CD11c
CD16
CD14
CD141
CD1c
FMO

Supplementary figure 1. Cellular composition of BAL

(A) Bar graph shows total BAL cells as concentration per litre. Graphs show mean ± SD. **(B)** Differential count of BAL cells from HC (n=30), non-LS (n=88) and LS patients (n=20). Graphs show the percentage (upper row) as well as the absolute numbers (lower row) of lymphocytes, macrophages/monocyte-derived cells, neutrophils, eosinophils, and basophils. Graphs show mean ± SD. **(C)** CD4/CD8 ratio in BAL as assessed by flow cytometry and staining against surface antigens to identify CD4 and CD8 positive. Graph shows the CD4/CD8 ratio in BAL as mean ± SD. HC n=30, non-LS n=81, and LS patients n=16. **(D)** Bar graphs show the percentage of alveolar macrophages as assessed by flow cytometry. SSC^{hi} and autofluorescence qualified for identification (see gating strategy figure 1B). Box plots show median ± IQR. HC n=6, non-LS n=17, and LS patients n=5. **(E)** Violin plot shows the frequency of HLA-DR+ lineage negative MNPs in blood and BAL (see gating strategy figure 1B). Dotted lines indicate median and 25th and 75th percentile. HC n=28, non-LS n=76, and LS patients n=16. Statistical analysis was performed using the non-parametric Kruskal-Wallis with Dunn´s test for correction of multiple comparisons. *p < 0.05, **p < 0.01, ***p < 0.001, ****p < 0.0001. **(F)** Fluorescence minus one (FMO) controls for HLA-DR, CD123, CD11c, CD14, CD16, CD1c, and CD141.

Figure S2

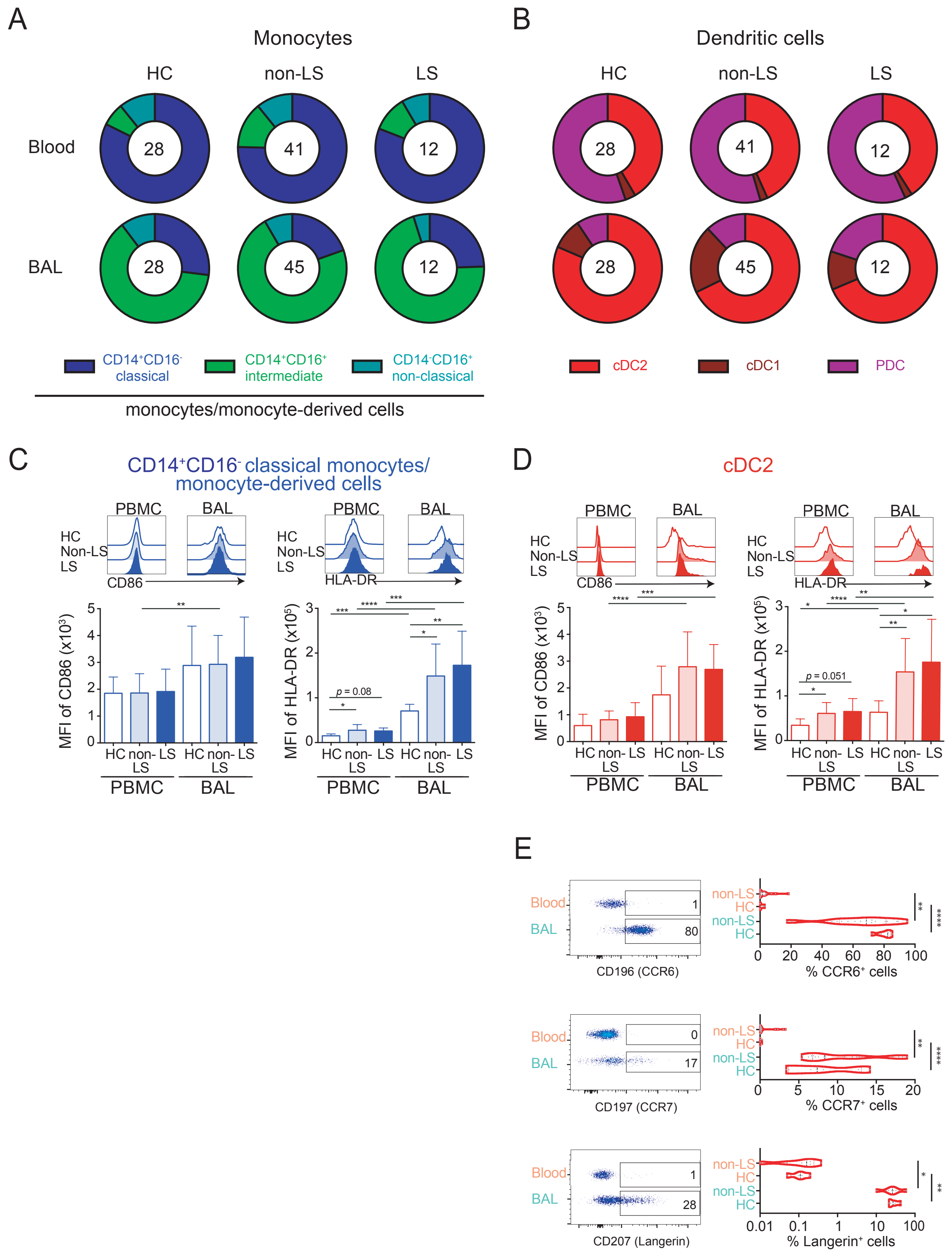
A
Monocytes
HC
non-LS
LS
Blood
28
41
12
BAL
28
45
12
CD14+CD16- classical
CD14+CD16+ intermediate
CD14-CD16+ non-classical
monocytes/monocyte-derived cells
B
Dendritic cells
HC
non-LS
LS
28
41
12
28
45
12
cDC2
cDC1
PDC
C
CD14+CD16- classical monocytes/ monocyte-derived cells
PBMC
BAL
HC
Non-LS
LS
CD86
HLA-DR
MFI of CD86 (x10³)
MFI of HLA-DR (x10⁵)
p = 0.08
HC non-LS LS HC non-LS LS
PBMC
BAL
D
cDC2
PBMC
BAL
HC
Non-LS
LS
CD86
HLA-DR
MFI of CD86 (x10³)
MFI of HLA-DR (x10⁵)
p = 0.051
HC non-LS LS HC non-LS LS
PBMC
BAL
E
Blood
BAL
1
80
CD196 (CCR6)
non-LS
HC
non-LS
HC
% CCR6+ cells
Blood
BAL
0
17
CD197 (CCR7)
% CCR7+ cells
Blood
BAL
1
28
CD207 (Langerin)
% Langerin+ cells

Supplementary figure 2. MNP frequencies and maturation differ between blood and BAL.

(A + B) Pie charts show the summary in distribution of different **(A)** monocytes/monocyte-derived cells and **(B)** DC subsets in blood and BAL of HC, non-LS and LS patients. Numbers inside the pie chart indicate the number of subjects. **(C + D)** Maturation of **(C)** $CD14^{+}CD16^{-}$ monocytes/monocyte-derived cells and **(D)** cDC2 were assessed by measuring the geometric mean fluorescence intensity of HLA-DR and CD86 in cells from blood and BAL of HC, non-LS and LS patients. Histogram show a representative example of HLA-DR and CD86 expression from a HC, non-LS patient and LS patient. Bar graphs show the summary as mean ± SD. Statistical analysis was performed using the non-parametric Kruskal-Wallis with Dunn´s test for correction of multiple comparisons. **(E)** Validation of differentially expressed genes for CCR6 (HC n=5, non-LS n=17), CCR7 (HC n=6, non-LS n=19), and CD207 (Langerin) (HC n=2, non-LS n=6) shown as an example in cDC2 from blood and BAL in HC and non-LS patients. Dotted lines indicate median, 25th and 75th percentile. Statistical analysis was performed using the non-parametric Mann-Whitney U unpaired t-test. *p < 0.05, **p < 0.01, ***p < 0.001 ****p < 0.0001.

Figure S3

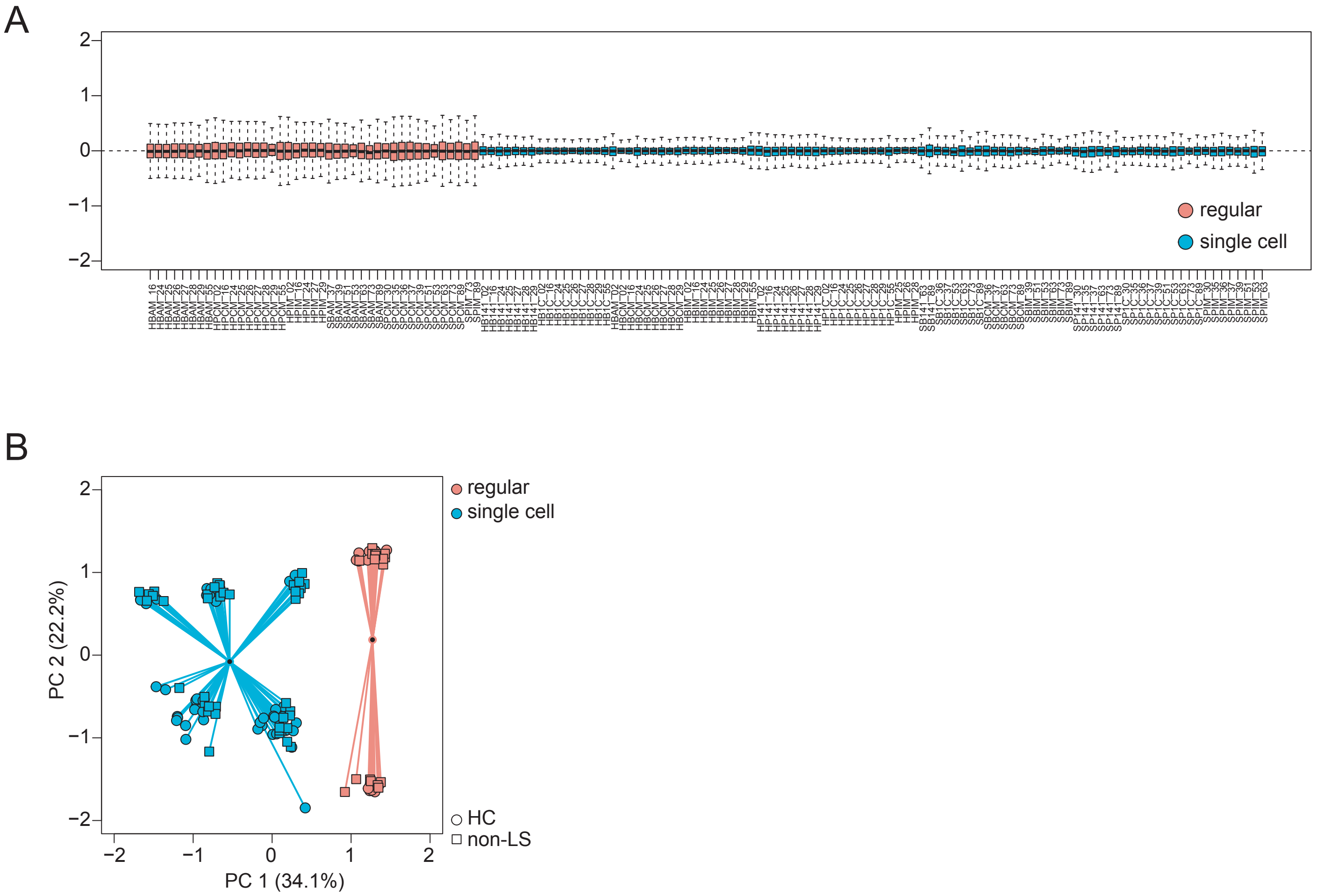
A
2
1
0
−1
−2
regular
single cell
B
regular
single cell
HC
non-LS
PC 2 (22.2%)
PC 1 (34.1%)
−2
−1
0
1
2

Supplementary figure 3. Impact of library preparation on gene expression.

(A) Relative log expression (RLE) plot for individual samples, grouped by library preparation method. **(B)** Principal component analysis of uncorrected normalised RNA-seq data, coloured and grouped by library preparation method.

Figure S4

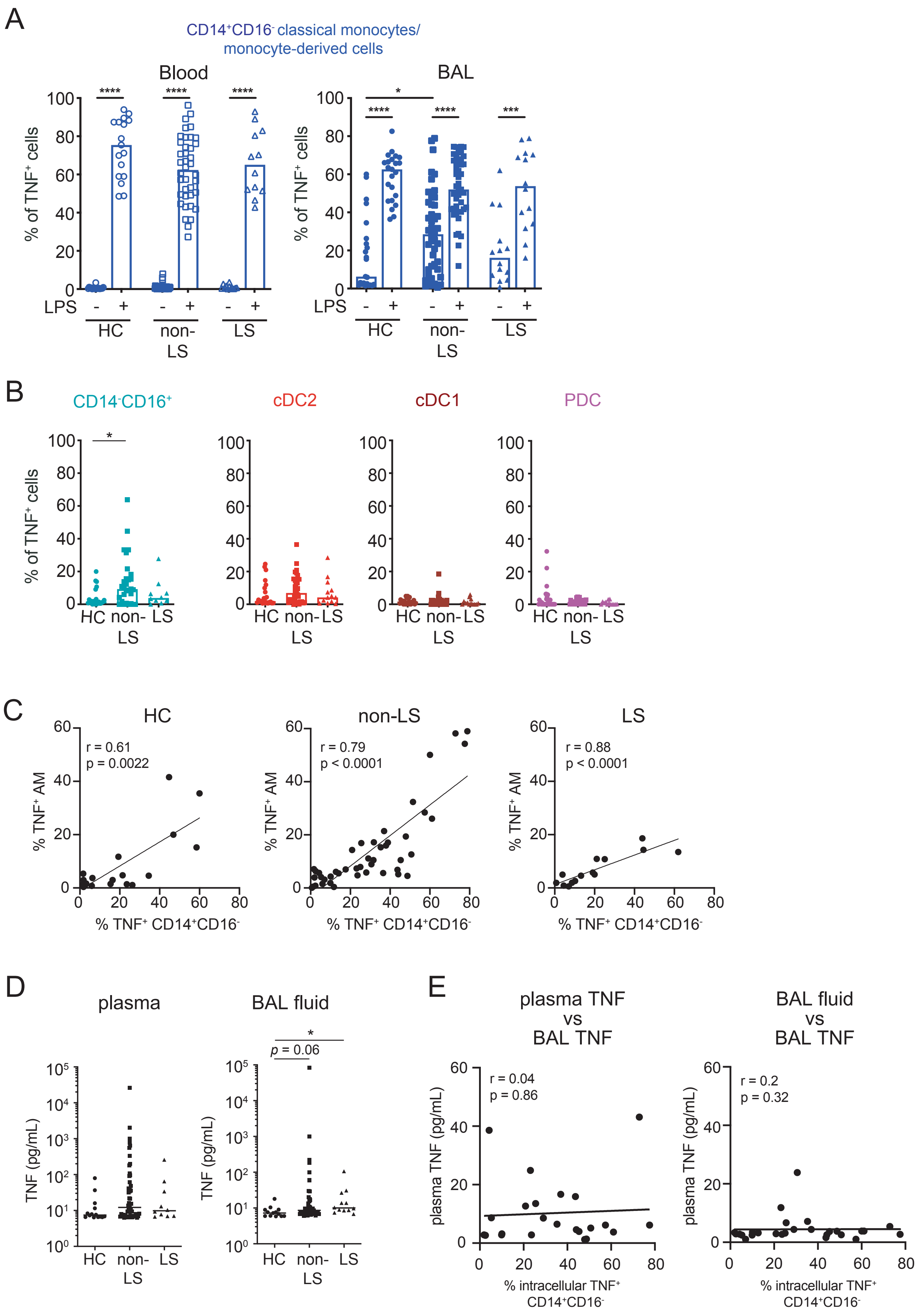

A
CD14+CD16- classical monocytes/ monocyte-derived cells
Blood
BAL
% of TNF+ cells
LPS - + - + - +
HC
non-LS
LS
B
CD14-CD16+
cDC2
cDC1
PDC
% of TNF+ cells
HC non-LS LS
C
HC
non-LS
LS
r = 0.61
p = 0.0022
r = 0.79
p < 0.0001
r = 0.88
p < 0.0001
% TNF+ AM
% TNF+ CD14+CD16-
D
plasma
BAL fluid
TNF (pg/mL)
p = 0.06
E
plasma TNF vs BAL TNF
BAL fluid vs BAL TNF
r = 0.04
p = 0.86
r = 0.2
p = 0.32
plasma TNF (pg/mL)
% intracellular TNF+ CD14+CD16-

Supplementary figure 4. Intracellular TNF expression in BAL MNPs do not correlate with soluble TNF in plasma or BAL fluid.

(A) Bar graphs show the frequency of TNF-expressing $CD14^{+}CD16^{-}$ monocytes/monocyte-derived cells from blood (open symbols) and BAL (filled symbols) of HC (circles), non-LS (squares) and LS patients (triangles) after 3h culture with and without LPS stimulation. Bars indicate the median. **(B)** BAL MNPs from HC, non-LS and LS patients were cultured for 3 hours in the presence of BFA and TNF expression determined by intracellular staining and analysis by flow cytometry. Bar graphs show the median. **(C)** Correlation of intracellular TNF expression in AM and $CD14^{+}CD16^{-}$ monocytes/monocyte-derived cells after 3h culture without stimulation in HC, non-LS and LS patients. r shows Spearman's correlation coefficient. **(D)** TNF concentration in plasma and BAL fluid of HC, non-LS and LS patients was measured using ELISA. BAL fluid was concentrated 20x prior to the ELISA. Graphs show individual values and line indicated the median. **(E)** Correlation of intracellular TNF expression by BAL $CD14^{+}CD16^{-}$ monocytes/monocyte-derived cells after 3h culture without stimulation and concentrations of soluble TNF in plasma or BAL fluid. r shows Spearman's correlation coefficient. Statistical analysis was performed using the non-parametric Kruskal-Wallis with Dunn´s test for correction of multiple comparisons. For correlation analysis the Spearman's rank test was used. $*p < 0.05$, $**p < 0.01$.

Figure S5

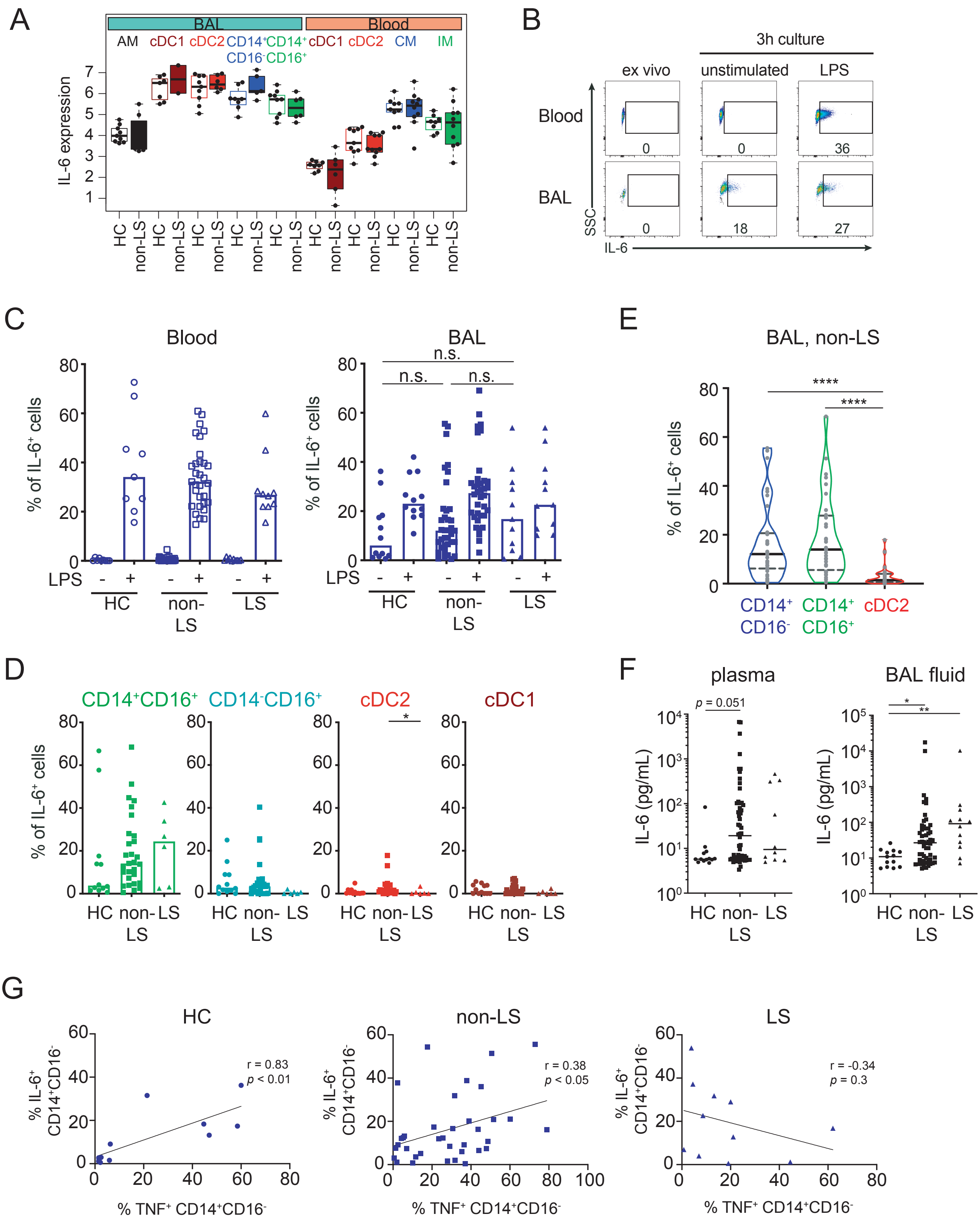

A
BAL
Blood
AM cDC1 cDC2 CD14+CD16- CD14+CD16+ cDC1 cDC2 CM IM
IL-6 expression
HC non-LS
B
3h culture
ex vivo
unstimulated
LPS
Blood
BAL
SSC
IL-6
0 0 36
0 18 27
C
Blood
BAL
% of IL-6+ cells
LPS - +
HC
non-LS
LS
n.s.
D
CD14+CD16+ CD14-CD16+ cDC2 cDC1
E
BAL, non-LS
****
CD14+ CD16- CD14+ CD16+ cDC2
F
plasma
BAL fluid
p = 0.051
IL-6 (pg/mL)
G
HC
non-LS
LS
% IL-6+ CD14+CD16-
% TNF+ CD14+CD16-
r = 0.83 p < 0.01
r = 0.38 p < 0.05
r = -0.34 p = 0.3

Supplementary figure 5. IL-6 expression by unstimulated pulmonary monocytes in sarcoidosis patients.

(A) Graph shows the relative IL-6 gene expression count from blood and BAL of the individual subsets in HC (open bars) and non-LS patients (filled bars). **(B)** Pseudocolour plots show IL-6 expression in $CD14^{+}CD16^{-}$ monocytes/monocyte-derived cells from blood and BAL of one representative non-LS patients stained *ex vivo* and after 3h culture without stimulation and after adding LPS (1ug/mL) in the presence of Brefeldin A (10ug/mL). **(C)** Bar graphs show the summary as frequency of IL-6 expression in $CD14^{+}CD16^{-}$ monocytes/monocyte-derived cells from blood and BAL of HC (circles, n=12), non-LS (squares, n=36) and LS patients (triangles, n=11) after 3h culture with and without LPS stimulation. Bars indicate the median. **(D)** BAL MNPs from HC, non-LS and LS patients were cultured for 3 hours in the presence of BFA and IL-6 expression determined by intracellular staining and analysis by flow cytometry. Bar graphs show the median. **(E)** Violin plots show a summary of IL-6 expression by BAL $CD14^{+}CD16^{-}$ and $CD14^{+}CD16^{+}$ monocytes/monocyte-derived cells and cDC2 from non-LS patients cultured for 3h without stimulation. Black lines indicate the median, 25th and 75th percentile. **(F)** IL-6 concentrations in plasma and BAL fluid of LS patients (plasma n=10, BAL fluid n=12), non-LS patients (plasma n=56, BAL fluid n=52) and HC (plasma and BAL fluid n=13) was assessed using ELISA. BAL fluid was 20x concentrated prior to the ELISA. **(G)** Graphs show the correlation of unstimulated TNF and IL-6 expression in $CD14^{+}CD16^{-}$ monocytes/monocyte-derived cells from BAL of HC (n=12), non-LS (n=11), and LS patients (n=36). r indicates Spearman's correlation coefficient. Statistical analyses were performed using the non-parametric Kruskal-Wallis test with Dunn's test for correction of multiple comparisons and Spearman's correlation analysis. *$p < 0.05$, **$p < 0.01$, ****$p < 0.0001$.

Figure S6

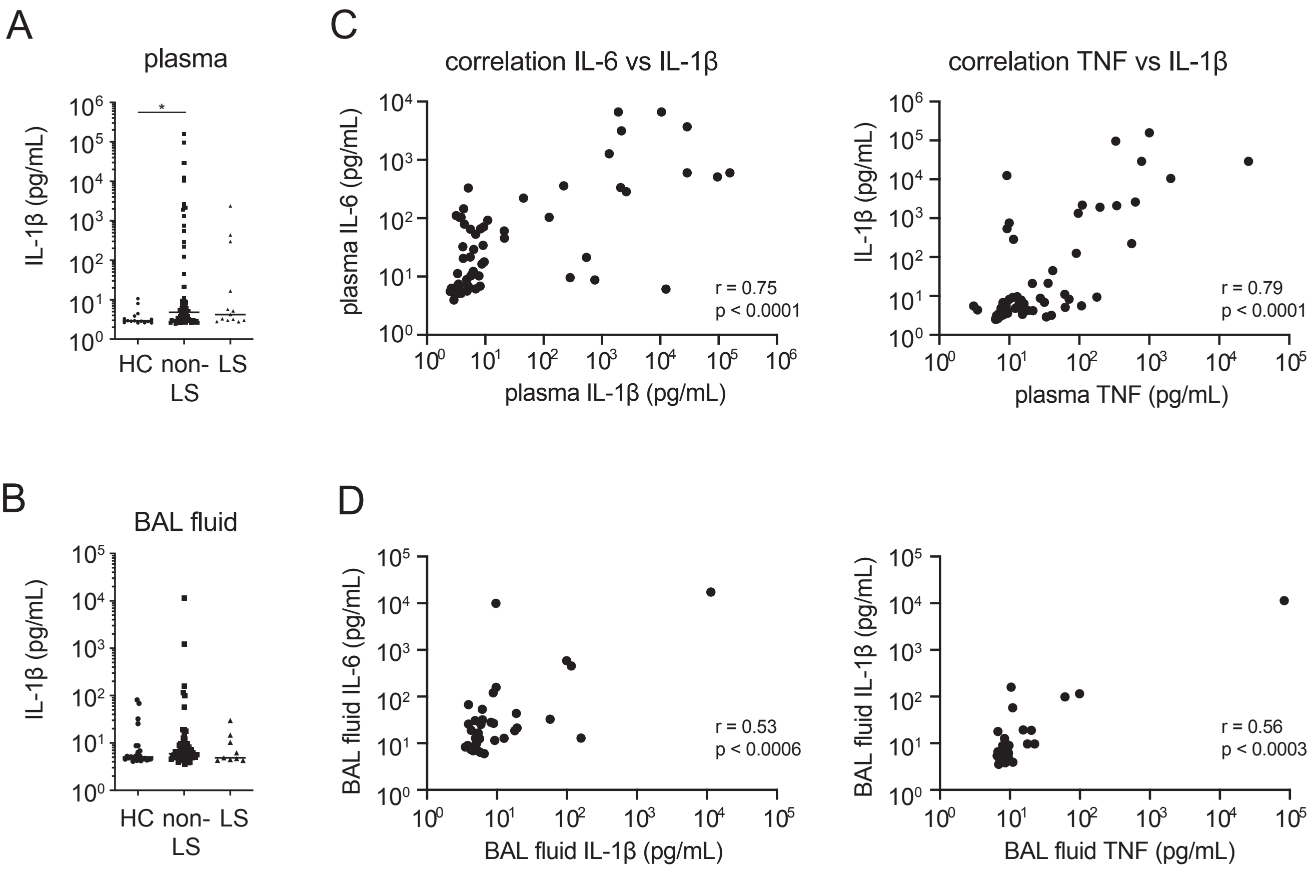

A
plasma
IL-1β (pg/mL)
HC
non-LS
LS
B
BAL fluid
IL-1β (pg/mL)
HC
non-LS
LS
C
correlation IL-6 vs IL-1β
plasma IL-6 (pg/mL)
plasma IL-1β (pg/mL)
r = 0.75
p < 0.0001
correlation TNF vs IL-1β
IL-1β (pg/mL)
plasma TNF (pg/mL)
r = 0.79
p < 0.0001
D
BAL fluid IL-6 (pg/mL)
BAL fluid IL-1β (pg/mL)
r = 0.53
p < 0.0006
BAL fluid IL-1β (pg/mL)
BAL fluid TNF (pg/mL)
r = 0.56
p < 0.0003

Supplementary figure 6. IL-1β expression in plasma and BAL fluid.

(A + B) IL-1β concentrations in serum and BAL fluid of healthy controls (HC), non-LS and LS patients. **(C + D)** Corelation of IL-1β and IL-6 or TNF concentration in plasma or BAL fluid of non-LS patients. HC: plasma n=15, BAL fluid n=24; non-LS: plasma n= 81, BAL fluid n=56; LS: plasma n=13, BAL fluid n=9. Statistical analysis was performed using the non-parametric Kruskal-Wallis test with Dunn's test for correction of multiple comparisons. *$p < 0.05$.

Figure S7

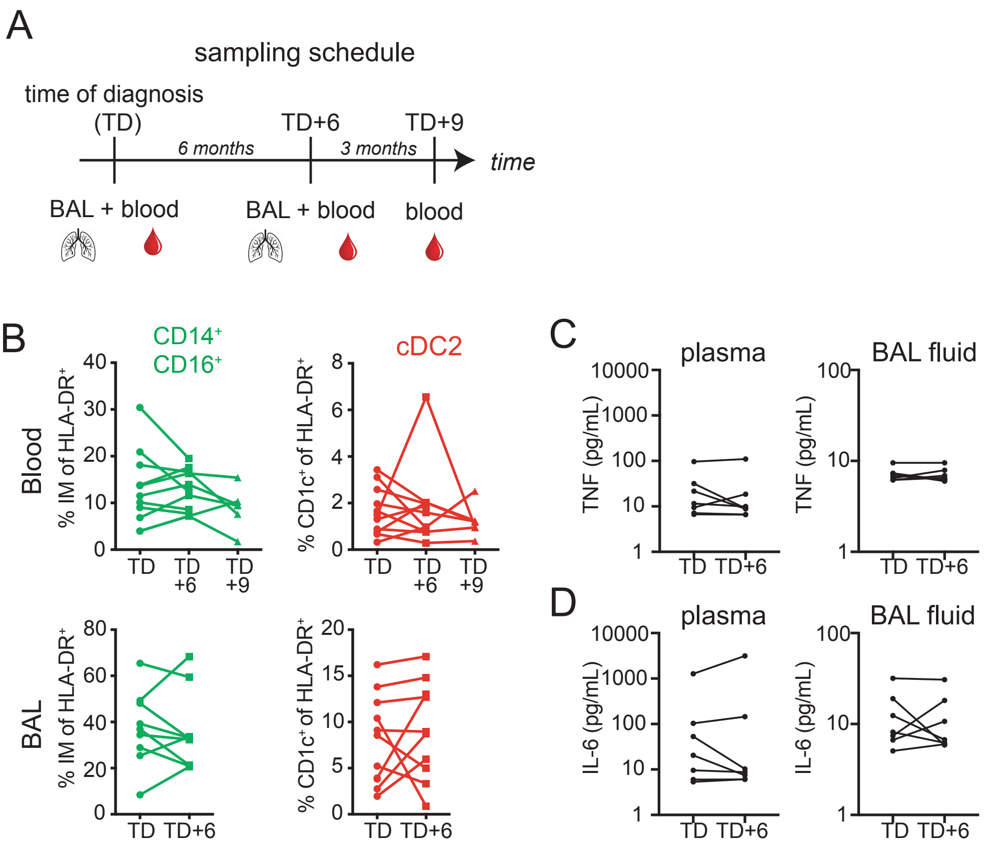
A
sampling schedule
time of diagnosis
(TD)
TD+6
TD+9
6 months
3 months
time
BAL + blood
BAL + blood
blood
B
CD14+
CD16+
cDC2
Blood
BAL
% IM of HLA-DR+
% CD1c+ of HLA-DR+
TD
TD +6
TD +9
TD TD+6
C
plasma
BAL fluid
TNF (pg/mL)
D
plasma
BAL fluid
IL-6 (pg/mL)

Supplementary figure 7. Frequencies of MNPs over time.

(A) A cohort of non-LS patients was included in a separate study that required the patients to undergo a first bronchoscopy at time of diagnosis (TD) to collect blood and BAL. A second bronchoscopy was performed 6 months later (TD + 6) to collect blood and BAL and a third time point another 3 months later (TD + 9) to collect blood only. **(B)** Frequencies of $CD14^{+}CD16^{+}$ monocytes/monocyte-derived cells and cDC2 in non-LS patients over the time course of 9 months in blood (upper row) and BAL (lower row). Lines connect individual patients. **(C + D)** Graphs show the concentrations of **(C)** TNF and **(D)** IL-6 measured in plasma and BAL fluid of the patients at time of diagnosis and after 6 months. Lines connect each individual. Statistical analyses were performed using the non-parametric paired Wilcoxon signed-rank test.

Figure S8

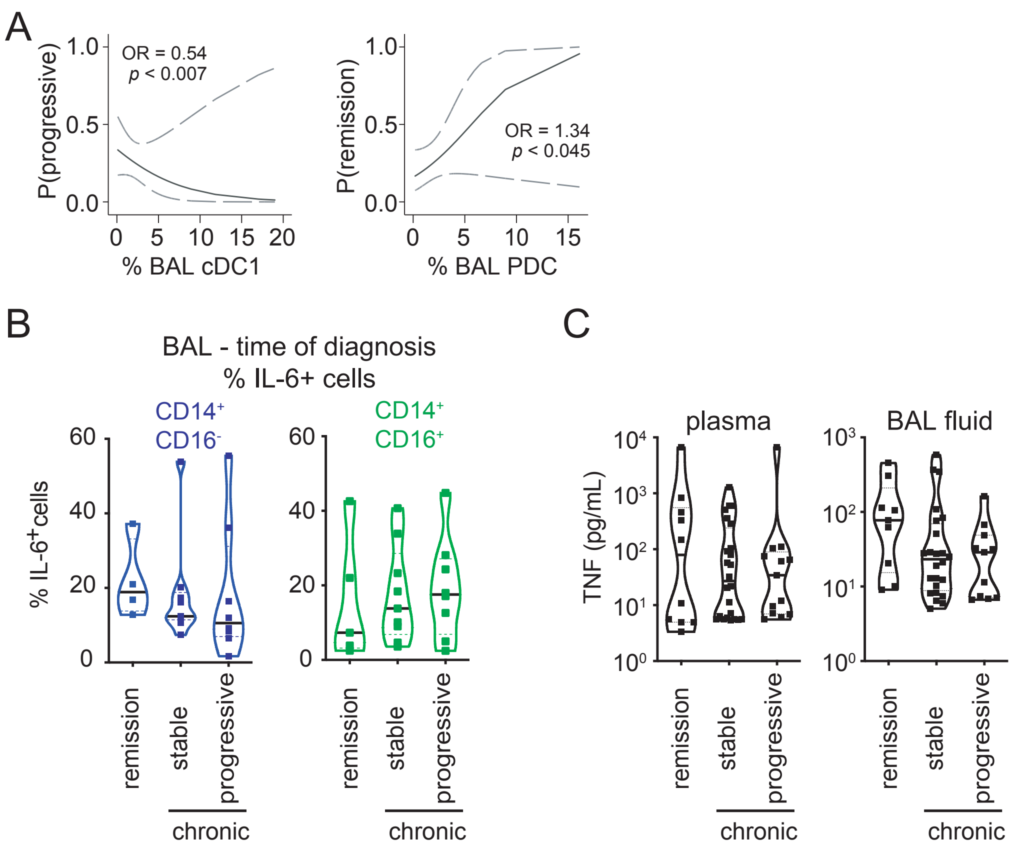
A
P(progressive)
OR = 0.54
p < 0.007
1.0
0.5
0.0
0 5 10 15 20
% BAL cDC1
P(remission)
OR = 1.34
p < 0.045
0 5 10 15
% BAL PDC
B
BAL - time of diagnosis
% IL-6+ cells
CD14+ CD16-
CD14+ CD16+
% IL-6+cells
60
40
20
0
remission
stable
progressive
chronic
C
plasma
BAL fluid
TNF (pg/mL)
10^4
10^3
10^2
10^1
10^0

Supplementary figure 8. DCs are predictors of disease severity in sarcoidosis.

(A) Plots show predictive modelling of frequencies of cDC1 and PDCs in BAL of sarcoidosis patients at time of diagnosis and probability for disease outcome. **(B + C)** Violin plots show **(B)** TNF expression after 3h culture without stimulation by $CD14^{+}CD16^{-}$ and $CD14^{+}CD16^{+}$ monocytes/monocyte-derived cells and AM at time of diagnosis and **(C)** TNF concentrations in plasma and BAL fluid and were grouped based on the outcome. Statistical analyses were performed using the non-parametric Kruskal-Wallis with Dunn's test for correction of multiple comparisons and predictive modelling using linear regression. *$p < 0.05$. OR: odds ratio.
