# Supplementary material for: Monocytes in sarcoidosis are potent tumour necrosis factor producers and predict disease outcome
Source: Eur Respir J. 2021 Jul 22;58(1):2003468. doi: 10.1183/13993003.03468-2020 (PMC8295505; doi:10.1183/13993003.03468-2020)
Supplement: Supplementary file 2 [file ERJ-03468-2020.Shareable.pdf]

Monocytes in sarcoidosis are potent tumour necrosis factor producers and predict disease outcome

SHAREABLE PDF

Rico Lepzien [1], Sang Liu[1], Paulo Czarnewski [2], Mu Nie[1], Björn Österberg[1], Faezzah Baharom [1], Jamshid Pourazar [3], Gregory Rankin [3], Anders Eklund [4,5], Matteo Bottai [6], Susanna Kullberg [4,5], Anders Blomberg [3], Johan Grunewald [4,5] and Anna Smed-Sörensen [1]

[1]Division of Immunology and Allergy, Dept of Medicine Solna, Karolinska Institutet, Karolinska University Hospital, Stockholm, Sweden. [2]Dept of Biochemistry and Biophysics, National Bioinformatics Infrastructure Sweden, Science for Life Laboratory, Stockholm University, Stockholm, Sweden. [3]Dept of Public Health and Clinical Medicine, Division of Medicine, Umeå University, Umeå, Sweden. [4]Division of Respiratory Medicine, Dept of Medicine Solna and Centre for Molecular Medicine, Karolinska Institutet, Karolinska University Hospital, Stockholm, Sweden. [5]Dept of Respiratory Medicine, Theme Inflammation and Infection, Karolinska University Hospital, Stockholm, Sweden. [6]Division of Biostatistics, Institute of Environmental Medicine, Karolinska Institutet, Stockholm, Sweden.

Corresponding author: Anna Smed-Sörensen (anna.smed.sorensen@ki.se)

Check for updates

Shareable abstract (@ERSpublications)
Phenotypic, transcriptomic and functional mapping of blood and pulmonary mononuclear phagocytes in sarcoidosis patients found that frequency and function of pulmonary monocytes at time of diagnosis predict 2-year outcome in sarcoidosis https://bit.ly/2JX8fhr

Cite this article as: Lepzien R, Liu S, Czarnewski P, *et al*. Monocytes in sarcoidosis are potent tumour necrosis factor producers and predict disease outcome. *Eur Respir J* 2021; 58: 2003468 [DOI: 10.1183/13993003.03468-2020].

This single-page version can be shared freely online.

Copyright ©The authors 2021

This version is distributed under the terms of the Creative Commons Attribution Licence 4.0.

This article has supplementary material available from erj.ersjournals.com

Received: 11 Sept 2020
Accepted: 17 Dec 2020

Abstract

Background Pulmonary sarcoidosis is an inflammatory disease characterised by granuloma formation and heterogeneous clinical outcome. Tumour necrosis factor (TNF) is a pro-inflammatory cytokine contributing to granuloma formation and high levels of TNF have been shown to associate with progressive disease. Mononuclear phagocytes (MNPs) are potent producers of TNF and highly responsive to inflammation. In sarcoidosis, alveolar macrophages have been well studied. However, MNPs also include monocytes/monocyte-derived cells and dendritic cells, which are poorly studied in sarcoidosis, despite their central role in inflammation.

Objective To determine the role of pulmonary monocyte-derived cells and dendritic cells during sarcoidosis.

Methods We performed in-depth phenotypic, functional and transcriptomic analysis of MNP subsets from blood and bronchoalveolar lavage (BAL) fluid from 108 sarcoidosis patients and 30 healthy controls. We followed the clinical development of patients and assessed how the repertoire and function of MNP subsets at diagnosis correlated with 2-year disease outcome.

Results Monocytes/monocyte-derived cells were increased in blood and BAL of sarcoidosis patients compared to healthy controls. Interestingly, high frequencies of blood intermediate monocytes at time of diagnosis associated with chronic disease development. RNA sequencing analysis showed highly inflammatory MNPs in BAL of sarcoidosis patients. Furthermore, frequencies of BAL monocytes/monocyte-derived cells producing TNF without exogenous stimulation at time of diagnosis increased in patients that were followed longitudinally. In contrast to alveolar macrophages, the frequency of TNF-producing BAL monocytes/monocyte-derived cells at time of diagnosis was highest in sarcoidosis patients that developed progressive disease.

Conclusion Our data show that pulmonary monocytes/monocyte-derived cells are highly inflammatory and can be used as a predictor of disease outcome in sarcoidosis patients.
